# Rejuvenated Autologous Adult Stem Cells: Emerging Front Runners in the Fight Against Aging and Associated Diseases

**DOI:** 10.3390/cells14151153

**Published:** 2025-07-25

**Authors:** An Yu, Changguo Ma, Min Hu

**Affiliations:** Yunnan Key Laboratory of Cell Therapy for Refractory Diseases, Kunming University, Kunming 650214, China; anyu@kmu.edu.cn (A.Y.); machangguo@kmu.edu.cn (C.M.)

**Keywords:** adult stem cells, autologous transplantation, cellular rejuvenation, cellular reprogramming

## Abstract

The growing global elderly population underscores the escalating importance of anti-aging interventions to combat age-related diseases and extend both health span and lifespan. Over the past decades, various anti-aging interventions have gained recognition, each with its unique set of advantages and limitations. Notably, the transplantation of rejuvenated autologous adult stem cells is standing out as a powerful strategy that holds significant promise in combating age-related functional decline and diseases. This review delves into our current biological insights into cellular rejuvenation and provides an overview of both pre-clinical and clinical experiences with autologous and allogeneic adult stem cell transplantations. It reinforces the concept that rejuvenated adult stem cells constitute a pivotal element in the quest for the fountain of youth. Additionally, we examine the technical challenges involved in obtaining and utilizing these rejuvenated adult stem cells.

## 1. Introduction

Embryonic stem cells (ESCs) are pluripotent stem cells derived from the inner cell mass of a blastocyst, an early-stage pre-implantation embryo. They have the unique ability to self-renew indefinitely in culture and differentiate into almost any cell type of the three germ layers: ectoderm, mesoderm, and endoderm. This makes them a promising source for studying early human development. Adult stem cells, also known as somatic stem cells, are multipotent stem cells found in various tissues throughout the body. They are responsible for maintaining and repairing their respective tissues. Unlike ESCs, adult stem cells are limited in both their differentiation and proliferative potential and typically give rise to cell types that are related to their tissue of origin.

The apparent immortality of hydra polyps, which seem to defy the aging process, can be attributed to one key factor: their adult stem cells possess an extraordinary ability to self-renew indefinitely [1]. Meanwhile, mammalian adult stem cell pool exhaustion is a hallmark of organism aging [2,3]. The replenishment of biologically youthful adult stem cells into aged organisms may offer compelling evidence that the exhaustion of adult stem cells is not only a hallmark of aging but also a pivotal cause of mammalian aging. Support for this hypothesis comes from studies where the transplantation of either young mesenchymal stem cells (MSCs) [4] or young hematopoietic stem cells (HSCs) [5] into old mice can delay aging and significantly extend the lifespan of old mice. In the past decade, numerous experimental studies have yielded insights, shedding light on the mechanisms behind the exhaustion of mammalian adult stem cells as organisms age. Among these insights, it seems that repeatedly activated adult stem cells caused mTORC1 (mammalian target of rapamycin complex 1) activation, which leads to adult stem cell premature differentiation, is a key molecular event resulting in adult stem cell exhaustion [6,7].

Adult stem cell transplantation is a straightforward method for replenishing the depleting stem cell reserves in aging organisms. In fact, numerous clinical practices have employed adult stem cell transplantations to treat various diseases and enhance the functionality of tissues and organs [8,9,10,11]. Both autologous and allogeneic stem cells can be used for stem cell transplantation. Transplanted allogeneic stem cells, owing to their inherent immunogenicity, can induce the activation of an immune response, which can lead to the death and clearance of the transplanted stem cells [12]. For autologous stem cell transplantation, the stem cells from an old organism are also aged, and it has been reported that the transplantation of old MSCs from aged mice is not able to extend the lifespan of old mice, whereas the transplantation of young MSCs can extend the lifespan of old mice [4]. Therefore, biological young or rejuvenated autologous adult stem cells are the key resources for replenishing exhausted adult stem cell pools to slow down the aging process and treat age-related diseases.

In this review, we provide a concise overview of the characteristics of current anti-aging interventions and try to elucidate the reasons why rejuvenated autologous adult stem cells are gaining prominence as a crucial tool against aging. We also address the limitations and challenges associated with obtaining and using rejuvenated autologous adult stem cells.

## 2. Aging and Currently Prevailing Anti-Aging Measures

Aging is a complex, time-dependent biological process characterized by progressive functional decline across multiple organ systems; increased vulnerability to disease and death due to accumulated molecular and cellular damage; and loss of homeostasis and reduced resilience to stress.

There have been many different theories that attempt to explain aging. For instance, the Free Radical Theory of aging posits that reactive oxygen species (ROS) cause cumulative damage to DNA, proteins, and lipids, leading to cellular dysfunction. Antioxidants (e.g., SOD enzymes) counteract this but decline with age [13]; the Hyperfunction theory of aging describes that aging stems from the inappropriate persistence or reactivation of developmental pathways (e.g., mTOR, insulin/IGF-1) beyond their beneficial early-life functions. This “hyperfunction” leads to pathological overgrowth, cellular stress, and organ damage [14], and most recently, the Information Theory of Aging: Aging is primarily caused by loss of epigenetic information, disrupting gene regulation. DNA damage repairs “distract” epigenetic regulators (e.g., SIRT1), preventing their return to original genomic sites. This creates a vicious cycle of dysregulation and further damage [15,16].

Currently, the major anti-aging strategies include caloric restriction, such as dietary restriction and intermittent fasting; the application of chemical compounds such as rapamycin, metformin, and nicotinamide mononucleotide (NMN); as well as cell-based anti-aging interventions, which mainly refer to the application of immune cells [17] and stem cells [4].

There are several limitations to using anti-aging compounds. For example, although rapamycin consistently extends mouse lifespan in several different studies [18,19,20,21,22], which suggests rapamycin is a potent anti-aging drug, rapamycin also induces obvious unwanted side effects, including fatty liver [23,24], hyperlipidemia, glucose intolerance [25], and immune suppression that increases the risk of infections [26]. Metformin mildly extends mouse lifespan [27]; however, metformin may increase the risk of lactic acidosis [28,29]. In addition, combining metformin and another anti-aging drug, SRT1 720, an activator of SIRT1, reduced mouse lifespan [30]. These compound-based anti-aging interventions require long-term, unintermittent drug treatment or at least a daily treatment within a period [18].

Heterochronic parabiosis has been reported to be able to rejuvenate some aging features of the old mice that were exposed to the young mice’s blood, and it was suggested that the hormone GDF11 (growth differentiation factor 11) mediated the rejuvenation effects [31,32]. However, the anti-aging effects of GDF11 have been severely questioned in the following studies [33,34].

Dietary restriction and intermittent fasting demand considerable willpower and discipline, particularly when individuals must endure the discomfort of hunger, which can be a significant barrier for many people. Consequently, it is uncommon for individuals to maintain a lifelong regimen of caloric restriction. Therefore, stem cell-based anti-aging interventions may offer a painless approach.

## 3. Very Small Embryonic-Like (VSEL) Stem Cells

Very Small Embryonic-Like (VSEL) stem cells are a rare population of pluripotent stem cells that have garnered significant attention in the field of regenerative medicine due to their unique characteristics and potential therapeutic applications. VSEL stem cells are defined as small, pluripotent stem cells that express markers characteristic of embryonic stem cells (ESCs), epiblast stem cells (EPI-SCs), and primordial germ cells (PGCs). These cells were first identified in murine bone marrow and later in human cord blood and peripheral blood [35,36]. VSELs are typically very small, ranging from 3 to 6 μm in diameter. They express a unique set of markers, including Sca-1, Lin(-), CD45(-), SSEA-4, and CXCR4, which distinguish them from other stem cell populations [35,37]. VSELs exhibit pluripotency, meaning they have the potential to differentiate into cells from all three germ layers (ectoderm, mesoderm, and endoderm). This capability has been demonstrated both in vitro and in vivo [38,39]. VSELs possess an epigenetic profile similar to that of embryonic stem cells, with partial DNA demethylation in regulatory regions of pluripotency and germ-line genes. This profile allows them to maintain a state of pluripotency while being sensitive to tissue-specific signals [37].

VSELs have shown promise in promoting tissue repair and regeneration. For instance, studies have demonstrated their ability to generate skeletal structures in vivo, indicating their potential for treating bone-related disorders [38]. Transplantation of expanded VSELs has been shown to improve left ventricular function and remodeling after myocardial infarction in animal models. This suggests that VSELs could be used to treat heart diseases by promoting cardiac tissue regeneration [40]. VSELs have also been explored for their potential in treating neurological disorders. Their ability to differentiate into neural lineages makes them a potential candidate for therapies targeting neurodegenerative diseases [39]. Given their pluripotency and potential for tissue rejuvenation, VSELs are considered a promising candidate for anti-aging therapies. They may play a role in the normal rejuvenation of adult tissues and could be harnessed to decelerate aging processes [36]. In summary, VSEL stem cells represent a promising avenue in regenerative medicine, with their unique pluripotency and differentiation capabilities offering potential solutions for a range of age-related and degenerative diseases.

## 4. Adult Stem Cell Pool Exhaustion Is Inevitable During Natural Aging

It has been well recognized that there are at least two different statuses of adult stem cells in vivo: activated stem cells and quiescent stem cells. Activated stem cells are typically in the cell cycle and exhibit high metabolic activity and biosynthetic capacity. They can rapidly respond to signals of tissue damage or regeneration, proliferating and differentiating to repair tissues. Quiescent stem cells are in a reversible G0 phase cell cycle arrest, distinct from the irreversible G0 arrest observed in terminally differentiated or senescent cells [41,42].

**Figure 1:**
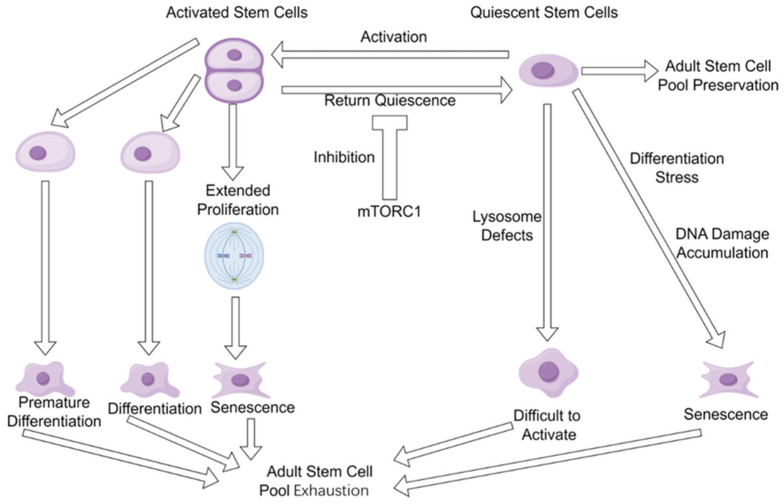
Adult stem cell pool exhaustion during aging. Both activated adult stem cells and quiescent adult stem cells have their own issues that drive the stem cell pool exhaustion during aging.

Activated adult stem cells play a vital role in supporting body growth during the developmental stages of neonates and juveniles. However, if these cells fail to re-enter a quiescent state during tissue repair and regeneration in adulthood, they may undergo premature differentiation, leading to exhaustion of the stem cell pool [7,43]. Furthermore, sustained cellular proliferation under such conditions can ultimately trigger senescence. Studies in muscle stem cells [43] and neural stem cells [44] provided evidence that entering the quiescent state prevented these adult stem cells from being fully depleted. However, aging brains use niche-derived inflammatory signals and the Wnt antagonist sFRP5 to induce neural stem cell quiescence, making quiescent neural stem cells more resistant to regenerating the injured brain [44]. One potential molecular mechanism underlying the impaired reactivation of aged quiescent neural stem cells in response to growth factor signaling is autophagy dysfunction, which is supported by evidence showing that treatment with the autophagy-promoting drug rapamycin significantly enhances the activation efficiency of these cells under growth factor stimulation [45]. In addition, it was revealed that DNA damage progressively accumulates in quiescent hematopoietic stem cells; however, the accumulated DNA damage will be efficiently repaired once the quiescent stem cells return to the cell cycle [46]. Thus, both activated and quiescent states of adult stem cells have distinct issues that collectively drive the exhaustion of the stem cell pool (Figure 1). Therefore, the replenishment of adult stem cells in aged animals/humans could be an efficient approach to prevent/ameliorate exhaustion of the stem cell pool by increasing adult stem cell numbers.

## 5. Adult Stem Cell Transplantation

Adult stem cell transplantation is a direct strategy to replenish exhausted stem cell pools in aging organisms. It has emerged as a promising therapeutic approach in numerous preclinical and clinical studies targeting specific pathologies, particularly age-related diseases, thereby highlighting its translational potential. For example, ischemic stroke is a typical age-related disease, and the transplantations of both mesenchymal stem cells (MSCs) and neural stem cells (NSCs) have been widely used to treat ischemic stroke in patients and animal models. In ischemic stroke rats, MSC transplantation resulted in smaller infarction size and improved neurological function scores [47], and corrected stroke-induced circuit disruptions even at the chronic stage [48]. In ischemic stroke patients, MSC transplantation resulted in improvement in the patients’ upper extremity and muscle strength, spasticity, and fine motor functions [49]. Transplantation of NSCs in ischemic stroke rats showed long-term sustained recovery of motor function, reduced infarction volume, improved local inflammatory environment, and angiogenesis [50].

In addition to ischemic stroke, NSC transplantation has been used to treat Alzheimer’s Disease (AD) model mice; the results showed that the transplanted neural stem cells restored spatial memory in AD model mice [51]. The transplanted neural stem cells can differentiate into multiple types of functional neurons and glial cells in AD mice [52]. There are numerous studies on adult stem cell transplantation to treat other diseases in both human patients and animal models. For example, transplantation of autologous HSCs in Autoimmune Diseases may help T cells recover [53].

A clinical trial investigated the safety and efficacy of intravenous administration of allogeneic mesenchymal stem cells (allo-hMSCs) in elderly patients with frailty [54]. The study enrolled 30 patients with a mean age of 75.5 ± 7.3 years and randomized them into three groups receiving either 100 million (100 M), 200 million (200 M) allo-hMSCs, or placebo. The primary endpoint was the incidence of treatment-emergent serious adverse events (TE-SAEs) at 1 month post-infusion, while secondary endpoints included physical performance, patient-reported outcomes, and immune markers of frailty measured at 6 months post-infusion. Key findings of this trial are as follows: 1. Safety: No therapy-related TE-SAEs were reported at 1 month post-infusion, indicating the safety of intravenous allo-hMSCs in individuals with aging frailty. 2. Physical Performance: The 100 M group showed significant improvements in physical performance measures, including the 6 min walk test (*p* = 0.01), short physical performance battery (*p* = 0.03), and forced expiratory volume in 1 s (*p* = 0.025). 3. Immune Markers: Both the 100 M- and 200 M-groups demonstrated improvements in immune markers, with decreased serum TNF-α levels (*p* = 0.03) and enhanced B cell intracellular TNF-α (*p* < 0.0001 for 100 M, *p* = 0.002 for 200 M). These results highlight the potential therapeutic benefits of allo-hMSCs in improving physical performance and reducing inflammation in elderly patients with frailty. The findings suggest that allo-hMSCs could be a promising intervention for addressing age-related frailty and associated conditions. Regarding the lack of similar trials since this study, several factors may contribute to this gap: 1. Regulatory Hurdles: The use of allogeneic stem cells in clinical trials involves complex regulatory requirements to ensure safety and efficacy. These include rigorous preclinical testing, manufacturing standards, and long-term follow-up protocols. 2. Ethical Considerations: The ethical implications of using allogeneic cells, including informed consent and potential risks, need to be carefully addressed in each trial. 3. Resource Intensity: Conducting such trials requires substantial resources, including funding, infrastructure, and expertise in stem cell therapy and clinical trial management. 4. Technical Challenges: Ensuring the viability and functionality of stored allogeneic cells over time is a significant technical challenge that needs to be overcome.

Although transplantation of both autologous and allogeneic adult stem cells has been widely used in pre-clinical and clinical studies, there is clear evidence that autologous stem cell transplantation possesses a significantly better performance than allogeneic stem cell transplantation. For instance, in a stroke model of mice, transplantation of autologous MSCs produced significant improvement (54–70%) in sensorimotor function, whereas allogeneic MSCs improved only 31.7% [55]. For human Knee Osteoarthritis, autologous MSCs also possess better effects than allogeneic MSCs [56]. Very recently, a case report has shown that autologous skin fibroblast-derived MSCs displayed much better therapeutic effects on treating Sjögren’s syndrome than allogeneic MSCs did [57]. These observations are reasonable because the immune system may eliminate or at least significantly reduce the transplanted allogeneic adult stem cells within a short period of time, restricting their functions to repair damaged tissues. However, for autologous adult stem cells, unless umbilical cord mesenchymal stem cells and hematopoietic stem cells derived from umbilical cord blood are cryopreserved in liquid nitrogen for long-term storage at birth, autologous stem cells will age along with the aging of the body. The concept of storing autologous biological materials at birth, such as Wharton’s jelly [58] and cord blood [59], has gained significant attention in the field of regenerative medicine due to its rich content of mesenchymal stem cells (MSCs) and hematopoietic stem cells (HSCs). These cells hold immense potential for future therapeutic applications, particularly in addressing age-related diseases and conditions. Wharton’s jelly, the gelatinous substance found in the umbilical cord, is a rich source of MSCs. These cells have demonstrated significant potential for tissue repair and regeneration due to their high proliferative capacity, multilineage differentiation potential, and immunomodulatory properties. Storing Wharton’s jelly at birth provides a unique opportunity to preserve a young and potent source of MSCs that can be utilized later in life for various therapeutic purposes. For instance, WJ-MSCs have shown promise in treating conditions such as osteoarthritis [60], cardiovascular diseases [61], and neurodegenerative disorders [62]. The autologous nature of these cells reduces the risk of immune rejection, making them a highly valuable resource for personalized medicine. Cord blood is another valuable source of HSCs [63], which are essential for the maintenance and repair of the hematopoietic system. Storing cord blood at birth ensures a readily available source of young and healthy HSCs that can be used for hematopoietic stem cell transplantation (HSCT) in the future. This is particularly important for conditions such as leukemia, lymphoma, and other blood disorders where HSCT is a critical treatment option. Additionally, the use of autologous CB-HSCs eliminates the risk of graft-versus-host disease (GVHD), a common complication associated with allogeneic HSCT [64]. While the potential benefits of storing Wharton’s jelly and cord blood are substantial, there are several challenges and considerations that need to be addressed. These include the cost of storage, the accessibility of these resources, and the long-term viability of the stored cells. Additionally, the regulatory and ethical considerations surrounding the use of these biological materials must be carefully managed to ensure their safe and effective application in clinical settings.

As mentioned above, transplanting aged MSCs has no anti-aging effect on the body [4]. Therefore, for elderly individuals who have missed the opportunity to preserve their young autologous stem cells at birth, obtaining biologically young autologous stem cells is currently quite challenging.

Cellular rejuvenation is an emerging concept that refers to the process by which aged cells regain the characteristics of biologically young cells. Over the past decade, numerous studies have focused on implementing cellular rejuvenation, offering a potential alternative for obtaining biologically young autologous adult stem cells for elderly individuals.

## 6. Dedifferentiation and Rejuvenation

The most well-known approach of cellular rejuvenation is the Yamanaka factor (OCT4, SOX2, KLF4, and c-MYC) based cellular reprogramming. By overexpressing these four genes, terminally differentiated somatic cells, such as skin fibroblasts, can be converted into embryonic stem cell-like cells, namely induced pluripotent stem cells (iPSCs). iPSCs are profoundly rejuvenated stem cells, of which epigenetic age is completely erased [65]; however, they cannot be directly used for transplantation because they form teratoma in vivo [66].

Dedifferentiation and rejuvenation are two important events that occur in the process of reprogramming differentiated somatic cells into pluripotent iPSCs. Here, we propose that cell dedifferentiation refers to the use of reprogramming methods to drive differentiated somatic cells or adult stem cells toward cell types earlier in the cell lineage of body development, which is usually evidenced by the acquisition of broader differentiation potential or stemness. To obtain the effects of rejuvenation and avoid full dedifferentiation that bears the risk of cancer, partial reprogramming was performed, in which the pluripotent factors (OCT4, SOX2, KLF4, and c-Myc) were only overexpressed for a short time (2–4 days) [67,68]. It was suggested that partial reprogramming leads to a reduction in the epigenetic age of cells before loss of somatic identity; thus, there could be a time window where rejuvenation can be achieved with a minimized risk of cancer [69]. In partial reprogramming, the rejuvenation effects are limited because pluripotent factors were overexpressed for only 2–4 days. For example, in human fibroblasts, 4-day overexpression of pluripotent factors (OCT4, SOX2, KLF4, c-MYC, LIN28, and NANOG) only reduced 3.4 years of DNA methylation age, while in human endothelial cells, 4-day overexpression of pluripotent factors reduced 4.94 years of DNA methylation age [68]. Unlike fully reprogrammed iPSCs, of which the rejuvenation effects can be kept for a very long time unless the iPSCs are differentiated, the rejuvenation effects of partially reprogrammed cells can only be maintained for a short period that is 4 days in premature aging mouse cells [67] and 4–6 days in human fibroblasts and endothelial cells [68].

**Figure 2:**
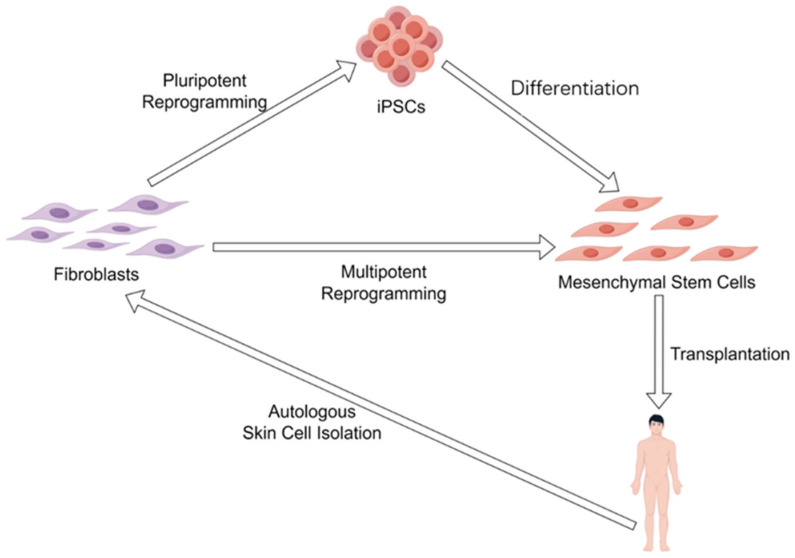
Different reprogramming strategies to obtain autologous mesenchymal stem cells. Mesenchymal stem cells can either be obtained by autologous iPSCs differentiation or autologous fibroblasts reprogramming (dedifferentiation) toward multipotency.

In addition to overexpressing the Yamanaka factors, combinations of chemicals have also been used to induce iPSCs from terminally differentiated somatic cells, such as skin fibroblasts [70]. Therefore, it should be reasonably expected that cellular rejuvenation effects can be obtained by using some combinations of chemicals [71]. In particular, chemically induced cellular reprogramming is a stepwise process, allowing for the generation of intermediate cellular stages that exhibit significant rejuvenation effects during the reprogramming process. Indeed, an intermediate plastic state, which was termed as limb-bud-like progenitors, was identified during chemically induced reprogramming [70] and captured by using a combination of Y27632, 616452 (Repsox), CHIR99021, SCG-CBP30, SAG, TTNBP, JNK-IN-8, and bFGF [72]. Notably, these limb-bud-like progenitors derived from fibroblasts showed strongly rejuvenated effects as evidenced by significantly down-regulated p21 and up-regulated Lamin B1, as well as the highly proliferative capacity, and displayed robust osteogenic and chondrogenic differentiation potential [72], which in turn is a sign of dedifferentiation. Identifying and capturing this intermediate plastic state during chemically induced reprogramming provides a notion that cellular rejuvenation effects can be acquired by reprogramming toward multipotency, and to gain cellular rejuvenation effects, reprogramming toward pluripotency is sufficient but not necessary. This concept was raised by Jacob C. Kimmel a few years ago [73], and the research he and his colleagues provided involved experimental evidence supporting this concept, which is that the multipotent gene *Msx1* overexpression partially restores youthful gene expression in aged myogenic cells [73]. Because multipotent stem cells, such as adult stem cells, are less primitive than pluripotent stem cells (e.g., iPSCs and ESCs), they may possess a minimized risk of forming malignant tumors; indeed, the chemically induced limb-bud-like progenitors have no tumorigenicity [72]. Therefore, using limited dedifferentiation to reprogram differentiated somatic cells to a multipotent state, such as that of adult stem cells, may offer a more advantageous approach for achieving rejuvenation effects compared to fully dedifferentiating somatic cells into iPSCs, especially when referring to clinical practices. For example, rejuvenated MSCs can be obtained from iPSCs [74]; Yet, in clinical settings, ensuring all MSCs from iPSCs are fully differentiated and free from undifferentiated iPSCs contamination remains challenging. While MSCs can also be obtained from fibroblasts by chemically induced reprogramming [75] without passing through the iPSCs stage, this provides a theoretically safer approach to gain autologous MSCs for patients and the elderly who need autologous MSC transplantation (Figure 2).

In addition to these reprogramming-based rejuvenation approaches, certain chemicals and interventions have demonstrated robust rejuvenation potential for adult stem cells. Aged neural stem cells (NSCs) can be functionally rejuvenated by simultaneously upregulating Plagl2 and inhibiting Dyrk1a [76]. For hematopoietic stem cells (HSCs), 2,3,5,4′-tetrahydroxystilbene-2-O-β-D-glucoside (TSG) [77], uridine [78], and small RhoGTPase Cdc42 inhibitor CASIN [79] were reported to rejuvenate HSCs efficiently. Notably, transplantation of rejuvenated HSCs by CASIN treatment into old mice significantly extends the lifespan of these old mice [79], indicating that rejuvenated HSCs display similar anti-aging functionality to the biologically youthful HSCs [5]. For mesenchymal stem cells (MSCs), FOXO3 double mutations (S253A/S345A), which make FOXO3 resistant to Akt phosphorylation and consistently present in the cell nucleus, confer MSCs with youthful traits, including enhanced self-renewal, reduced senescence-associated beta-galactosidase activity, extended telomeres, and improved heterochromatin stability [80]. Importantly, transplantation of these FOXO3 double-site mutated MSCs into older cynomolgus monkeys systemically improved their aging phenotypes, such as decelerating multi-organ aging clocks, improved brain function, improved bone density, and better reproductive health [80]. A microRNA named miR-302b that specifically targeted the cell cycle inhibitors Cdkn1a and Ccng2 was reported to be a key rejuvenation molecule in vivo [81]. Also, it was reported that knockdown of AP2A1 reversed senescence-associated phenotypes, exhibiting features of cellular rejuvenation, while its overexpression in young cells advanced senescence phenotypes [82]. The anti-aging effects of young/rejuvenated adult stem cell transplantations in experimental animals were summarized in Table 1. These successful rejuvenation experiments across various types of adult stem cells demonstrate that cellular rejuvenation can be effectively achieved through artificial interventions. Moreover, the transplantation of these rejuvenated adult stem cells represents a powerful anti-aging strategy in aged organisms.

The above-mentioned rejuvenation interventions could raise a series of important questions. For instance, is there a core subset of gene regulatory networks that are shared by different rejuvenation interventions in different types of adult stem cells or somatic cells? Do the rejuvenation events during early embryogenesis use the same gene regulatory networks to make sure that every newborn life is youthful [83,84]? Are all the different rejuvenation interventions closely coupled with cell dedifferentiation? What are the molecular relationships between rejuvenation and dedifferentiation? Further investigations in cellular rejuvenation may help to answer these questions in the future.

## 7. Challenges of Gaining and Using Rejuvenated Adult Stem Cells

Despite some successful advancements in cellular rejuvenation, researchers and clinicians still face significant challenges in obtaining rejuvenated adult stem cells suitable for direct clinical use. Several critical issues hinder the development and application of clinically viable rejuvenated adult stem cells.

First, so far, there is still a lack of a golden standard that determines the degree of rejuvenation. The most ideal rejuvenation is that rejuvenated aged adult stem cells are physiologically identical to those youthful adult stem cells in young individuals. There have been some aging/rejuvenation indicators used to assess cellular rejuvenation effects, such as H3K9me3 and H4K20me3 levels, as well as DNA damage marker γH2AX [67], DNA methylation clock [68], or telomere length [80]. The epigenetic clock is a molecular biomarker of aging based on DNA methylation levels at specific CpG sites across the genome. This method was first introduced by Steve Horvath in 2013 [85] and has since been refined and expanded to include multiple tissue types and species [86]. The epigenetic clock provides a highly accurate measure of biological age, often referred to as “epigenetic age,” which correlates strongly with chronological age and predicts mortality and age-related diseases more effectively than chronological age alone. Telomere length is another widely used biomarker of cellular aging. Telomeres, the protective caps at the ends of chromosomes, shorten with each cell division, and critically short telomeres can lead to cellular senescence or apoptosis. Telomere length has been extensively studied as a marker of biological age [87] and is associated with various age-related diseases. There are some limitations to using telomere length to estimate biological age: Variable Rate of Shortening: The rate of telomere shortening can vary significantly among individuals and cell types, making it less reliable as a universal biomarker of aging [88]. While telomere length is associated with aging, it does not predict mortality as strongly as the epigenetic clock. Some studies have shown that individuals with longer telomeres can still experience age-related diseases [89]. In comparison, the epigenetic clock offers several advantages over telomere length as a biomarker of biological age. Its high accuracy, predictive power, and versatility make it a superior tool for estimating cellular aging and evaluating rejuvenation interventions. While telomere length remains an important biomarker, its limitations in terms of variability and predictive power suggest that the epigenetic clock is a more reliable and comprehensive measure of biological age.

However, even a combination of these indicators can not determine whether rejuvenated cells are similar or identical to authentic young cells, especially when there is no conclusion about how each indicator contributes to the rejuvenation degree.

Second, how long can the rejuvenation effects be kept? The rejuvenation effects of 4-day Yamanaka factor overexpressing cells can only be maintained for 4 days in premature aging mouse cells after stopping the overexpression of Yamanaka factors [67]. The rejuvenated limb-bud-like progenitors are captured and need to be kept in a combination of Y27632, 616452 (Repsox), CHIR99021, SCG-CBP30, SAG, TTNBP, JNK-IN-8, and bFGF medium [72], yet it is still unknown whether the rejuvenation effects can be long-term maintained in common basic medium, such as low glucose DMEM with 8–10% fetal bovine serum (FBS). After all, after intravenous injection, there would be no additional chemicals that can help to maintain the rejuvenation effects of these stem cells in vivo. We speculate that to gain profound rejuvenation effects, an epigenetic barrier needs to be eliminated during the reprogramming process, and in turn, the eliminated epigenetic barrier needs to be re-established to maintain the acquired rejuvenation effects before they disappear.

Third, other safety issues need to be carefully assessed in addition to tumorigenicity. For example, intravenous injection of large doses of allogeneic MSCs induced a series of symptoms of respiratory failure and heart failure through inducing disseminated intravascular coagulation, exhaustion of platelets, and coagulation factors [90].

## 8. Summary

The global rise in aging populations underscores an urgent need for effective interventions against age-related functional decline and diseases. While current strategies—including caloric restriction, pharmacological agents (e.g., rapamycin, metformin), and heterochronic parabiosis—offer partial benefits, they face significant limitations such as side effects, poor long-term adherence, and unresolved safety concerns. Central to organismal aging is the exhaustion of adult stem cell pools, driven by repeated activation-induced mTORC1 signaling, premature differentiation, and impaired quiescence. Transplantation studies demonstrate that replenishing aged organisms with biologically young stem cells mitigates aging phenotypes and extends lifespan. Critically, autologous transplants outperform allogeneic approaches by evading immune rejection, yet sourcing youthful autologous cells from aged individuals remains a fundamental challenge.

Cellular rejuvenation emerges as a transformative solution. Techniques like partial reprogramming (transient Yamanaka factor expression) and chemical induction (e.g., generating limb-bud-like multipotent progenitors) reset epigenetic clocks without pluripotency-associated tumor risks. These approaches yield transiently rejuvenated cells with restored proliferative capacity, reduced senescence markers, and lineage-specific differentiation potential. Notably, transplanting rejuvenated hematopoietic or mesenchymal stem cells extends lifespan in aged mice and improves multi-organ function in primates. Rejuvenated autologous adult stem cells represent a paradigm shift in anti-aging therapy, merging the immunological advantages of autografts with the vitality of youth. Overcoming current limitations will cement their role as the pivotal frontier in extending healthspan and combating age-related diseases.

## Figures and Tables

**Table 1:** Young/rejuvenated adult stem cell transplantation improves aging phenotypes in experimental animals.

Young/Rejuvenated Stem Cells	Experimental Animals	Effects of Transplantation	Reference
Young Mesenchymal Stem Cells	mouse	slow the loss of bone density, and prolong the lifespan of old mice.	[4]
Young Hematopoietic Stem Cells	mouse	reductions in frailty measures and increases in food intake and body weight of aged recipients, and extend the lifespan of old mice.	[5]
Rejuvenated Hematopoietic Stem Cells	mouse	Enhance lymphoid and regenerative capacity in serial transplantation assays, and extend the healthspan and lifespan of aged mice.	[79]
FOXO3 double-mutated senescence-resistant human mesenchymal progenitor cells	macaque	a systemic reduction in aging indicators, such as cellular senescence, chronic inflammation, and tissue degeneration, without any detected adverse effects. Enhanced brain architecture and cognitive function, and alleviated the reproductive system decline.	[80]

## References

[1] Boehm A.M., Khalturin K., Anton-Erxleben F., Hemmrich G., Klostermeier U.C., Lopez-Quintero J.A., Oberg H.H., Puchert M., Rosenstiel P., Wittlieb J. (2012). FoxO is a critical regulator of stem cell maintenance in immortal Hydra. Proc. Natl. Acad. Sci. USA.

[2] López-Otín C., Blasco M.A., Partridge L., Serrano M., Kroemer G. (2013). The hallmarks of aging. Cell.

[3] López-Otín C., Blasco M.A., Partridge L., Serrano M., Kroemer G. (2023). Hallmarks of aging: An expanding universe. Cell.

[4] Shen J., Tsai Y.T., Dimarco N.M., Long M.A., Sun X., Tang L. (2011). Transplantation of mesenchymal stem cells from young donors delays aging in mice. Sci. Rep..

[5] Guderyon M.J., Chen C., Bhattacharjee A., Ge G., Fernandez R.A., Gelfond J.A.L., Gorena K.M., Cheng C.J., Li Y., Nelson J.F. (2020). Mobilization-based transplantation of young-donor hematopoietic stem cells extends lifespan in mice. Aging Cell.

[6] Feng R., Wu S., Li R., Huang K., Zeng T., Zhou Z., Zhong X., Songyang Z., Liu F. (2023). mTORC1-Induced Bone Marrow-Derived Mesenchymal Stem Cell Exhaustion Contributes to the Bone Abnormalities in Klotho-Deficient Mice of Premature Aging. Stem Cells Dev..

[7] Haller S., Kapuria S., Riley R.R., O’Leary M.N., Schreiber K.H., Andersen J.K., Melov S., Que J., Rando T.A., Rock J. (2017). mTORC1 Activation during Repeated Regeneration Impairs Somatic Stem Cell Maintenance. Cell Stem Cell.

[8] Bose G., Atkins H.L., Bowman M., Freedman M.S. (2019). Autologous hematopoietic stem cell transplantation improves fatigue in multiple sclerosis. Mult. Scler..

[9] Genchi A., Brambilla E., Sangalli F., Radaelli M., Bacigaluppi M., Furlan R., Andolfo A., Drago D., Magagnotti C., Scotti G.M. (2023). Neural stem cell transplantation in patients with progressive multiple sclerosis: An open-label, phase 1 study. Nat. Med..

[10] Ghobrial G.M., Anderson K.D., Dididze M., Martinez-Barrizonte J., Sunn G.H., Gant K.L., Levi A.D. (2017). Human Neural Stem Cell Transplantation in Chronic Cervical Spinal Cord Injury: Functional Outcomes at 12 Months in a Phase II Clinical Trial. Neurosurgery.

[11] Izadi M., Sadr Hashemi Nejad A., Moazenchi M., Masoumi S., Rabbani A., Kompani F., Hedayati Asl A.A., Abbasi Kakroodi F., Jaroughi N., Mohseni Meybodi M.A. (2022). Mesenchymal stem cell transplantation in newly diagnosed type-1 diabetes patients: A phase I/II randomized placebo-controlled clinical trial. Stem Cell Res. Ther..

[12] Wang Y., Tian M., Wang F., Heng B.C., Zhou J., Cai Z., Liu H. (2019). Understanding the Immunological Mechanisms of Mesenchymal Stem Cells in Allogeneic Transplantation: From the Aspect of Major Histocompatibility Complex Class I. Stem Cells Dev..

[13] Ziada A.S., Smith M.R., Côté H.C.F. (2020). Updating the Free Radical Theory of Aging. Front. Cell Dev. Biol..

[14] Blagosklonny M.V. (2022). Rapamycin treatment early in life reprograms aging: Hyperfunction theory and clinical practice. Aging.

[15] Lu Y.R., Tian X., Sinclair D.A. (2023). The Information Theory of Aging. Nat. Aging.

[16] Yang J.H., Hayano M., Griffin P.T., Amorim J.A., Bonkowski M.S., Apostolides J.K., Salfati E.L., Blanchette M., Munding E.M., Bhakta M. (2023). Loss of epigenetic information as a cause of mammalian aging. Cell.

[17] Amor C., Feucht J., Leibold J., Ho Y.J., Zhu C., Alonso-Curbelo D., Mansilla-Soto J., Boyer J.A., Li X., Giavridis T. (2020). Senolytic CAR T cells reverse senescence-associated pathologies. Nature.

[18] Bitto A., Ito T.K., Pineda V.V., LeTexier N.J., Huang H.Z., Sutlief E., Tung H., Vizzini N., Chen B., Smith K. (2016). Transient rapamycin treatment can increase lifespan and healthspan in middle-aged mice. Elife.

[19] Harrison D.E., Strong R., Sharp Z.D., Nelson J.F., Astle C.M., Flurkey K., Nadon N.L., Wilkinson J.E., Frenkel K., Carter C.S. (2009). Rapamycin fed late in life extends lifespan in genetically heterogeneous mice. Nature.

[20] Miller R.A., Harrison D.E., Astle C.M., Baur J.A., Boyd A.R., de Cabo R., Fernandez E., Flurkey K., Javors M.A., Nelson J.F. (2011). Rapamycin, but not resveratrol or simvastatin, extends life span of genetically heterogeneous mice. J. Gerontol. A Biol. Sci. Med. Sci..

[21] Miller R.A., Harrison D.E., Astle C.M., Fernandez E., Flurkey K., Han M., Javors M.A., Li X., Nadon N.L., Nelson J.F. (2014). Rapamycin-mediated lifespan increase in mice is dose and sex dependent and metabolically distinct from dietary restriction. Aging Cell.

[22] Neff F., Flores-Dominguez D., Ryan D.P., Horsch M., Schröder S., Adler T., Afonso L.C., Aguilar-Pimentel J.A., Becker L., Garrett L. (2013). Rapamycin extends murine lifespan but has limited effects on aging. J. Clin. Investig..

[23] Chaveroux C., Eichner L.J., Dufour C.R., Shatnawi A., Khoutorsky A., Bourque G., Sonenberg N., Giguère V. (2013). Molecular and genetic crosstalks between mTOR and ERRα are key determinants of rapamycin-induced nonalcoholic fatty liver. Cell Metab..

[24] Ge C., Ma C., Cui J., Dong X., Sun L., Li Y., Yu A. (2023). Rapamycin suppresses inflammation and increases the interaction between p65 and IκBα in rapamycin-induced fatty livers. PLoS ONE.

[25] Houde V.P., Brûlé S., Festuccia W.T., Blanchard P.G., Bellmann K., Deshaies Y., Marette A. (2010). Chronic rapamycin treatment causes glucose intolerance and hyperlipidemia by upregulating hepatic gluconeogenesis and impairing lipid deposition in adipose tissue. Diabetes.

[26] Shi G., Ozog S., Torbett B.E., Compton A.A. (2018). mTOR inhibitors lower an intrinsic barrier to virus infection mediated by IFITM3. Proc. Natl. Acad. Sci. USA.

[27] Martin-Montalvo A., Mercken E.M., Mitchell S.J., Palacios H.H., Mote P.L., Scheibye-Knudsen M., Gomes A.P., Ward T.M., Minor R.K., Blouin M.J. (2013). Metformin improves healthspan and lifespan in mice. Nat. Commun..

[28] DeFronzo R., Fleming G.A., Chen K., Bicsak T.A. (2016). Metformin-associated lactic acidosis: Current perspectives on causes and risk. Metabolism.

[29] Lalau J.D. (2010). Lactic acidosis induced by metformin: Incidence, management and prevention. Drug Saf..

[30] Palliyaguru D.L., Minor R.K., Mitchell S.J., Palacios H.H., Licata J.J., Ward T.M., Abulwerdi G., Elliott P., Westphal C., Ellis J.L. (2020). Combining a High Dose of Metformin With the SIRT1 Activator, SRT1720, Reduces Life Span in Aged Mice Fed a High-Fat Diet. J. Gerontol. A Biol. Sci. Med. Sci..

[31] Loffredo F.S., Steinhauser M.L., Jay S.M., Gannon J., Pancoast J.R., Yalamanchi P., Sinha M., Dall’Osso C., Khong D., Shadrach J.L. (2013). Growth differentiation factor 11 is a circulating factor that reverses age-related cardiac hypertrophy. Cell.

[32] Sinha M., Jang Y.C., Oh J., Khong D., Wu E.Y., Manohar R., Miller C., Regalado S.G., Loffredo F.S., Pancoast J.R. (2014). Restoring systemic GDF11 levels reverses age-related dysfunction in mouse skeletal muscle. Science.

[33] Egerman M.A., Cadena S.M., Gilbert J.A., Meyer A., Nelson H.N., Swalley S.E., Mallozzi C., Jacobi C., Jennings L.L., Clay I. (2015). GDF11 Increases with Age and Inhibits Skeletal Muscle Regeneration. Cell Metab..

[34] Smith S.C., Zhang X., Zhang X., Gross P., Starosta T., Mohsin S., Franti M., Gupta P., Hayes D., Myzithras M. (2015). GDF11 does not rescue aging-related pathological hypertrophy. Circ. Res..

[35] Kucia M., Wysoczynski M., Ratajczak J., Ratajczak M.Z. (2008). Identification of very small embryonic like (VSEL) stem cells in bone marrow. Cell Tissue Res..

[36] Ratajczak M.Z., Zuba-Surma E.K., Shin D.M., Ratajczak J., Kucia M. (2008). Very small embryonic-like (VSEL) stem cells in adult organs and their potential role in rejuvenation of tissues and longevity. Exp. Gerontol..

[37] Kim Y., Jeong J., Kang H., Lim J., Heo J., Ratajczak J., Ratajczak M.Z., Shin D.M. (2014). The molecular nature of very small embryonic-like stem cells in adult tissues. Int. J. Stem Cells.

[38] Havens A.M., Shiozawa Y., Jung Y., Sun H., Wang J., McGee S., Mishra A., Taichman L.S., Danciu T., Jiang Y. (2013). Human very small embryonic-like cells generate skeletal structures, in vivo. Stem Cells Dev..

[39] Havens A.M., Sun H., Shiozawa Y., Jung Y., Wang J., Mishra A., Jiang Y., O’Neill D.W., Krebsbach P.H., Rodgerson D.O. (2014). Human and murine very small embryonic-like cells represent multipotent tissue progenitors, in vitro and in vivo. Stem Cells Dev..

[40] Zuba-Surma E.K., Guo Y., Taher H., Sanganalmath S.K., Hunt G., Vincent R.J., Kucia M., Abdel-Latif A., Tang X.L., Ratajczak M.Z. (2011). Transplantation of expanded bone marrow-derived very small embryonic-like stem cells (VSEL-SCs) improves left ventricular function and remodelling after myocardial infarction. J. Cell. Mol. Med..

[41] de Morree A., Rando T.A. (2023). Regulation of adult stem cell quiescence and its functions in the maintenance of tissue integrity. Nat. Rev. Mol. Cell Biol..

[42] Urbán N., Cheung T.H. (2021). Stem cell quiescence: The challenging path to activation. Development.

[43] Kim J.H., Han G.C., Seo J.Y., Park I., Park W., Jeong H.W., Lee S.H., Bae S.H., Seong J., Yum M.K. (2016). Sex hormones establish a reserve pool of adult muscle stem cells. Nat. Cell Biol..

[44] Kalamakis G., Brüne D., Ravichandran S., Bolz J., Fan W., Ziebell F., Stiehl T., Catalá-Martinez F., Kupke J., Zhao S. (2019). Quiescence Modulates Stem Cell Maintenance and Regenerative Capacity in the Aging Brain. Cell.

[45] Leeman D.S., Hebestreit K., Ruetz T., Webb A.E., McKay A., Pollina E.A., Dulken B.W., Zhao X., Yeo R.W., Ho T.T. (2018). Lysosome activation clears aggregates and enhances quiescent neural stem cell activation during aging. Science.

[46] Beerman I., Seita J., Inlay M.A., Weissman I.L., Rossi D.J. (2014). Quiescent hematopoietic stem cells accumulate DNA damage during aging that is repaired upon entry into cell cycle. Cell Stem Cell.

[47] Liu Y., Zhao Y., Min Y., Guo K., Chen Y., Huang Z., Long C. (2022). Effects and Mechanisms of Bone Marrow Mesenchymal Stem Cell Transplantation for Treatment of Ischemic Stroke in Hypertensive Rats. Int. J. Stem Cells.

[48] Klein B., Ciesielska A., Losada P.M., Sato A., Shah-Morales S., Ford J.B., Higashikubo B., Tager D., Urry A., Bombosch J. (2025). Modified human mesenchymal stromal/stem cells restore cortical excitability after focal ischemic stroke in rats. Mol. Ther..

[49] Ercelen N., Karasu N., Kahyaoglu B., Cerezci O., Akduman R.C., Ercelen D., Erturk G., Gulay G., Alpaydin N., Boyraz G. (2023). Clinical experience: Outcomes of mesenchymal stem cell transplantation in five stroke patients. Front. Med..

[50] Wang P., Liu P., Ding Y., Zhang G., Wang N., Sun X., Li M., Li M., Bao X., Chen X. (2024). Transplantation of human neural stem cells repairs neural circuits and restores neurological function in the stroke-injured brain. Neural Regen. Res..

[51] Chen K.S., Noureldein M.H., McGinley L.M., Hayes J.M., Rigan D.M., Kwentus J.F., Mason S.N., Mendelson F.E., Savelieff M.G., Feldman E.L. (2023). Human neural stem cells restore spatial memory in a transgenic Alzheimer’s disease mouse model by an immunomodulating mechanism. Front. Aging Neurosci..

[52] Ji Q., Lv Y., Hu B., Su Y., Shaikh I.I., Zhu X. (2024). Study on the therapeutic potential of induced neural stem cells for Alzheimer’s disease in mice. Biol. Res..

[53] Santana-Gonçalves M., De Santis P.B., Malmegrim K.C.R., Oliveira M.C. (2025). T-cell Recovery After Autologous Hematopoietic Stem Cell Transplantation in Autoimmune Diseases. Adv. Exp. Med. Biol..

[54] Tompkins B.A., DiFede D.L., Khan A., Landin A.M., Schulman I.H., Pujol M.V., Heldman A.W., Miki R., Goldschmidt-Clermont P.J., Goldstein B.J. (2017). Allogeneic Mesenchymal Stem Cells Ameliorate Aging Frailty: A Phase II Randomized, Double-Blind, Placebo-Controlled Clinical Trial. J. Gerontol. Ser. A Biomed. Sci. Med. Sci..

[55] Myers M.I., Hines K.J., Gray A., Spagnuolo G., Rosenwasser R., Iacovitti L. (2025). Intracerebral Transplantation of Autologous Mesenchymal Stem Cells Improves Functional Recovery in a Rat Model of Chronic Ischemic Stroke. Transl. Stroke Res..

[56] Hsu C.C., Cheng J.H., Wang C.J., Ko J.Y., Hsu S.L., Hsu T.C. (2020). Shockwave Therapy Combined with Autologous Adipose-Derived Mesenchymal Stem Cells Is Better than with Human Umbilical Cord Wharton’s Jelly-Derived Mesenchymal Stem Cells on Knee Osteoarthritis. Int. J. Mol. Sci..

[57] Ma Z.X., Wu X.F., Cao L., Jiao C.Y., Ma D.P., Zhao Y.H., Yang Z.X., Hu M. (2025). Regenerative fibroblasts derived from autologous skin tissue for the treatment of Sjögren’s syndrome: A case report. Front. Immunol..

[58] Doi H., Kitajima Y., Luo L., Yan C., Tateishi S., Ono Y., Urata Y., Goto S., Mori R., Masuzaki H. (2016). Potency of umbilical cord blood- and Wharton’s jelly-derived mesenchymal stem cells for scarless wound healing. Sci. Rep..

[59] Warwick R., Armitage S. (2004). Cord blood banking. Best Pract. Res. Clin. Obstet. Gynaecol..

[60] Liang H., Suo H., Wang Z., Feng W. (2020). Progress in the treatment of osteoarthritis with umbilical cord stem cells. Hum. Cell.

[61] Attar A., Farjoud Kouhanjani M., Hessami K., Vosough M., Kojuri J., Ramzi M., Hosseini S.A., Faghih M., Monabati A. (2023). Effect of once versus twice intracoronary injection of allogeneic-derived mesenchymal stromal cells after acute myocardial infarction: BOOSTER-TAHA7 randomized clinical trial. Stem Cell Res. Ther..

[62] Wu K.J., Yu S.J., Chiang C.W., Lee Y.W., Yen B.L., Tseng P.C., Hsu C.S., Kuo L.W., Wang Y. (2018). Neuroprotective Action of Human Wharton’s Jelly-Derived Mesenchymal Stromal Cell Transplants in a Rodent Model of Stroke. Cell Transplant..

[63] Jahan S., Kaushal R., Pasha R., Pineault N. (2021). Current and Future Perspectives for the Cryopreservation of Cord Blood Stem Cells. Transfus. Med. Rev..

[64] Lombardo G., Lechanteur C., Briquet A., Seidel L., Willems E., Servais S., Baudoux E., Kerre T., Zachee P., Herman J. (2024). Co-infusion of mesenchymal stromal cells to prevent GVHD after allogeneic hematopoietic cell transplantation from HLA-mismatched unrelated donors after reduced-intensity conditioning: A double-blind randomized study and literature review. Stem Cell Res. Ther..

[65] Wang M., Lemos B. (2019). Ribosomal DNA harbors an evolutionarily conserved clock of biological aging. Genome Res..

[66] Abad M., Mosteiro L., Pantoja C., Cañamero M., Rayon T., Ors I., Graña O., Megías D., Domínguez O., Martínez D. (2013). Reprogramming in vivo produces teratomas and iPS cells with totipotency features. Nature.

[67] Ocampo A., Reddy P., Martinez-Redondo P., Platero-Luengo A., Hatanaka F., Hishida T., Li M., Lam D., Kurita M., Beyret E. (2016). In Vivo Amelioration of Age-Associated Hallmarks by Partial Reprogramming. Cell.

[68] Sarkar T.J., Quarta M., Mukherjee S., Colville A., Paine P., Doan L., Tran C.M., Chu C.R., Horvath S., Qi L.S. (2020). Transient non-integrative expression of nuclear reprogramming factors promotes multifaceted amelioration of aging in human cells. Nat. Commun..

[69] Olova N., Simpson D.J., Marioni R.E., Chandra T. (2019). Partial reprogramming induces a steady decline in epigenetic age before loss of somatic identity. Aging Cell.

[70] Guan J., Wang G., Wang J., Zhang Z., Fu Y., Cheng L., Meng G., Lyu Y., Zhu J., Li Y. (2022). Chemical reprogramming of human somatic cells to pluripotent stem cells. Nature.

[71] Yang J.H., Petty C.A., Dixon-McDougall T., Lopez M.V., Tyshkovskiy A., Maybury-Lewis S., Tian X., Ibrahim N., Chen Z., Griffin P.T. (2023). Chemically induced reprogramming to reverse cellular aging. Aging.

[72] Zhu J., Zhong X., He H., Cao J., Zhou Z., Dong J., Li H., Zhang A., Lyu Y., Li C. (2024). Generation of human expandable limb-bud-like progenitors via chemically induced dedifferentiation. Cell Stem Cell.

[73] Roux A.E., Zhang C., Paw J., Zavala-Solorio J., Malahias E., Vijay T., Kolumam G., Kenyon C., Kimmel J.C. (2022). Diverse partial reprogramming strategies restore youthful gene expression and transiently suppress cell identity. Cell Syst..

[74] Spitzhorn L.S., Megges M., Wruck W., Rahman M.S., Otte J., Degistirici Ö., Meisel R., Sorg R.V., Oreffo R.O.C., Adjaye J. (2019). Human iPSC-derived MSCs (iMSCs) from aged individuals acquire a rejuvenation signature. Stem Cell Res. Ther..

[75] Lai P.L., Lin H., Chen S.F., Yang S.C., Hung K.H., Chang C.F., Chang H.Y., Lu F.L., Lee Y.H., Liu Y.C. (2017). Efficient Generation of Chemically Induced Mesenchymal Stem Cells from Human Dermal Fibroblasts. Sci. Rep..

[76] Kaise T., Fukui M., Sueda R., Piao W., Yamada M., Kobayashi T., Imayoshi I., Kageyama R. (2022). Functional rejuvenation of aged neural stem cells by Plagl2 and anti-Dyrk1a activity. Genes Dev..

[77] Gao D., Yi W.W., Liu B., Zhang C.E., Yang C.C., Zeng L., Li L., Luo G., Zhang L., Ju Z.Y. (2025). Tetrahydroxy stilbene glucoside rejuvenates aging hematopoietic stem cells with predilection for lymphoid differentiation via AMPK and Tet2. J. Adv. Res..

[78] Zeng X., Shi C., Han Y., Hu K., Li X., Wei C., Ding L., Cui J., Huang S., Xu Y. (2024). A metabolic atlas of blood cells in young and aged mice identifies uridine as a metabolite to rejuvenate aged hematopoietic stem cells. Nat. Aging.

[79] Montserrat-Vazquez S., Ali N.J., Matteini F., Lozano J., Zhaowei T., Mejia-Ramirez E., Marka G., Vollmer A., Soller K., Sacma M. (2022). Transplanting rejuvenated blood stem cells extends lifespan of aged immunocompromised mice. NPJ Regen. Med..

[80] Lei J., Xin Z., Liu N., Ning T., Jing Y., Qiao Y., He Z., Jiang M., Yang Y., Zhang Z. (2025). Senescence-resistant human mesenchymal progenitor cells counter aging in primates. Cell.

[81] Bi Y., Qiao X., Cai Z., Zhao H., Ye R., Liu Q., Gao L., Liu Y., Liang B., Liu Y. (2025). Exosomal miR-302b rejuvenates aging mice by reversing the proliferative arrest of senescent cells. Cell Metab..

[82] Chantachotikul P., Liu S., Furukawa K., Deguchi S. (2025). AP2A1 modulates cell states between senescence and rejuvenation. Cell. Signal..

[83] Kerepesi C., Gladyshev V.N. (2023). Intersection clock reveals a rejuvenation event during human embryogenesis. Aging Cell.

[84] Kerepesi C., Zhang B., Lee S.G., Trapp A., Gladyshev V.N. (2021). Epigenetic clocks reveal a rejuvenation event during embryogenesis followed by aging. Sci. Adv..

[85] Horvath S. (2013). DNA methylation age of human tissues and cell types. Genome Biol..

[86] Haghani A., Li C.Z., Robeck T.R., Zhang J., Lu A.T., Ablaeva J., Acosta-Rodríguez V.A., Adams D.M., Alagaili A.N., Almunia J. (2023). DNA methylation networks underlying mammalian traits. Science.

[87] Daios S., Anogeianaki A., Kaiafa G., Kontana A., Veneti S., Gogou C., Karlafti E., Pilalas D., Kanellos I., Savopoulos C. (2022). Telomere Length as a Marker of Biological Aging: A Critical Review of Recent Literature. Curr. Med. Chem..

[88] Demanelis K., Jasmine F., Chen L.S., Chernoff M., Tong L., Delgado D., Zhang C., Shinkle J., Sabarinathan M., Lin H. (2020). Determinants of telomere length across human tissues. Science.

[89] Wikgren M., Karlsson T., Nilbrink T., Nordfjäll K., Hultdin J., Sleegers K., Van Broeckhoven C., Nyberg L., Roos G., Nilsson L.G. (2012). APOE ε4 is associated with longer telomeres, and longer telomeres among ε4 carriers predicts worse episodic memory. Neurobiol. Aging.

[90] Liao L., Shi B., Chang H., Su X., Zhang L., Bi C., Shuai Y., Du X., Deng Z., Jin Y. (2017). Heparin improves BMSC cell therapy: Anticoagulant treatment by heparin improves the safety and therapeutic effect of bone marrow-derived mesenchymal stem cell cytotherapy. Theranostics.

